# Reliability of repeated exposure to the human elevated plus-maze in virtual reality: Behavioral, emotional, and autonomic responses

**DOI:** 10.3758/s13428-022-02046-5

**Published:** 2022-12-21

**Authors:** Sarah V. Biedermann, Lateefah Roth, Daniel Biedermann, Johannes Fuss

**Affiliations:** 1 Social and Emotional Neuroscience Group, Department of Psychiatry and Psychotherapy, Center of Psychosocial Medicine, University Medical Center Hamburg-Eppendorf, Hamburg, Germany; 2 Institute of Forensic Psychiatry and Sex Research, Center for Translational Neuro- and Behavioral Sciences, University of Duisburg-Essen, Essen, Germany; 3 Human Behavior Laboratory, Institute for Sex Research, Sexual Medicine and Forensic Psychiatry, Center of Psychosocial Medicine, University Medical Center Hamburg-Eppendorf, Hamburg, Germany; 4 DIPF Leibniz Institute for Research and Information in Education, Frankfurt am Main, Germany

**Keywords:** Sensitization, Habituation, Mixed reality, Approach, Avoidance

## Abstract

Approach–avoidance conflicts are a hallmark of anxiety-related behaviors. A gold standard for assessing anxiety-related behaviors in rodents is the elevated plus-maze (EPM), which was recently translated to humans using immersive virtual reality. Repeated behavioral testing is particularly interesting for clinical and pharmacological research in humans but could be limited by habituation effects. Here, we tested whether comparable strategies that are used in rodents (different environments and inter-trial interval of 28 days) are sufficient to avoid habituation or sensitization effects on the EPM, making it possible to perform repeated measurement of anxiety-related behavior in humans. Moreover, we developed two novel virtual environments for repeated testing to explore whether a scenario resembling the real world is superior to a video game-like EPM in terms of lifelike physiological, emotional, and behavioral responses. On a behavioral level, no significant differences but a high correlation between first and repeated exposure to the human EPM independent of EPM version were found. On a psychophysiological level, salivary alpha-amylase, skin-conductance, and respiratory frequency increased at first and second exposure independent of EPM version. However, at repeated exposure, skin-conductance and heart rate showed indicators for anticipatory anxiety and a small sensitization effect, while no effect of real-world resemblance on these physiological measures was found. This was also reflected in slightly higher subjective anxiety levels at second exposure, although subjective anxiety still correlated strongly between first and second exposure. In conclusion, the human EPM can be used for longitudinal assessments of human anxiety-related behavior when strategies to avoid habituation and sensitization are considered.

## Introduction

Conventional approaches to studying human fear and anxiety-related behaviors are limited. They either use previously learned fear responses through classical conditioning (LeDoux, 1998) or reduce the human reaction to an artificial response of negative or positive tendencies, such as approach and avoidance, in a computer-based setup using eye or joystick movements (e.g., Blanchard, 2017). In doing so, conventional approaches do not take the complexity of real-life fear- and anxiety-related behavior into account (Kisker, Lange, et al., 2021a). Likewise, commonly used self-reports represent unreliable parameters for capturing anxiety-related behavior (LeDoux et al., 2017), since actual behavior often does not correlate reliably with subjective perception or memory of behavior (Zinbarg, 1998). Thus, anxiety-related behavior can occur without people explicitly recognizing that a fear-associated stimulus is present and without subjectively feeling anxious (Bertini et al., 2013; Mineka and Öhman, 2002; Tamietto and de Gelder, 2010; Whalen, 2004). Virtual reality (VR) has been shown to offer a true-to-life, rich and interactive tool (Felnhofer et al., 2015), which enables the creation of virtual environments (VE) that can trigger a variety of positive (relaxation: Baños et al., 2008, 2012, Riva et al., 2007, Serrano et al., 2013; joy: Baños et al., 2008, 2012; Felnhofer et al., 2015) and negative (boredom: Felnhofer et al., 2015; anger: Felnhofer et al., 2015; sadness: Baños et al., 2004; anxiety: Toet et al., 2009, Felnhofer et al., 2015, Riva et al., 2007; fear: Kisker, Lange, et al., 2021a) emotional states (see for review: Bernardo et al., 2020). A key feature of affect in VR is the relatively high immersivity and sense of presence in VR (Baños et al., 2004; Felnhofer et al., 2015; Slater & Wilbur, 1997) compared to conventional designs in psychological experimental research (Kisker, Lange, et al., 2021a; Schöne et al., 2021). The latter are believed not to elicit emotions but rather memories of emotions or re-experiencing of participants to induce affect (Harmon-Jones, 2019; Schöne et al., 2021). In contrast, VR allows for a “first person perspective” (Schöne et al., 2021) and a reduced meta-awareness of being in a simulation (Kisker et al., 2019b; Pan & Hamilton, 2018; Schöne et al., 2021). This allows participants to feel directly affected by their surrounding in a virtual environment (Schöne et al., 2021) and evokes authentic physiological and behavioral responses (Kisker, Lange, et al., 2021a; Schöne et al., 202). Often, VEs have been kept as realistic as possible by creating naturalistic scenarios such as parks (Baños et al., 2004, 2008; Felnhofer et al., 2015; Riva et al., 2007) or village squares and alleys (Toet et al., 2009). It has been suggested that realism is an important factor for VR simulations to elicit affect (Kisker, Lange, et al., 2021a; Schöne et al., 2021).

Approach–avoidance conflicts promise reliable and objective assessments; they have thus become a hallmark for anxiety-related behaviors (LeDoux et al., 2017). With the advances of novel mixed-reality paradigms, frightening (e.g., a scary cave, Kisker, Lange, et al., 2021a) or unpleasant (e.g., a visit to the dentist, Schöne et al., 2021) situations have been used to design VEs to study participants’ approach and avoidance tendencies (Biedermann et al., 2017; Kisker, Lange, et al., 2021a; Schöne et al., 2021). Studies have shown that during VR exposure, approach and avoidance motivations measured with an electroencephalogram expressed as frontal alpha asymmetries (FAAs) differed from FAAs obtained in conventional 2D setups (Schöne et al., 2021). It was suggested that current models of FAA interpretation are inconsistent with measures of approach and avoidance motivations under VR conditions because these models were based on simple artificial responses in a 2D setup, whereas VR elicits a more realistic and complex fear- and anxiety-related response at multiple levels (Kisker, Lange, et al., 2021a). VR could represent a tool to investigate more realistic cognitive and emotional underpinnings of anxiety-related behaviors as participants exhibit lifelike behaviors in an ecologically valid environment rather than responding to images (Schöne et al., 2021). Accordingly, participants in fear/anxiety-inducing VEs exhibited distinctly fear-related body language, such as moving more slowly and cautiously in the face of potential danger (Biedermann et al., 2017; Kisker et al., 2019a; Kisker, Gruber, & Schöne, 2021b; Kisker, Lange, et al., 2021a) or screaming, closing their eyes, and dodging (Lin, 2017). Thus, approach and avoidance in VR could be studied more reliably using behavioral and physiological parameters as used in standardized behavioral tests of animal models of anxiety research (Biedermann et al., 2017). The backward translation of well-established behavioral tasks from rodents to humans (Krakauer et al., 2017) allows for objective and standardized observation of authentic behavior in clinically relevant situations and thus greatly improves the diagnosis and study of behavioral characteristics in anxiety disorders. The elevated plus-maze (EPM) is one of the most extensively studied approach–avoidance conflict tasks, with more than 9400 scientific articles citing the EPM on PubMed in 2022. It is used in rodents primarily to assess the effects of novel substances, genetic factors, and environmental conditions on anxiety-related behaviors. Our research group developed a human version of the EPM using a virtual reality setup. We were able to demonstrate face, content, concurrent, predictive, and construct validity of the human EPM (Biedermann et al., 2017). Moreover, we found that pharmacological manipulation of the GABAergic and noradrenergic systems changed human behavior in the same direction as rodent behavior on the EPM (Biedermann et al., 2017). Interestingly, the open field test, another rodent task to assess approach–avoidance behavior, was also backward-translated from animals to humans on a football field and revealed validity of core behavioral markers across species (Walz et al., 2016). This test was recently established in virtual reality (Gromer et al., 2021). It can be assumed that neuroscience and psychiatry may benefit profoundly from translational behavioral tasks for discovering common brain–behavior relationships. Moreover, these translational tasks may be useful for clinical research to study the effects of psychotropic medication or psychotherapeutic interventions on approach–avoidance behaviors. In particular, in the early stages of researching novel compounds that are efficient in changing rodent behavior, it may be useful to see whether these molecules alter human behavior in the same direction on the same tasks. For this purpose, it would be methodologically beneficial to repeatedly measure behavior on a given task (e.g., before and after an intervention), provided the task shows stable test–retest profiles. However, applying the same test repeatedly in the same individual may be complicated by habituation or sensitization. Habituation is defined as a “behavioral response decrement” that results from repeated exposure to a given task and that does “not involve sensory adaptation/sensory fatigue or motor fatigue” (Rankin et al., 2009, p. 136). Some responses that underlie long-term habituation first show an increase in responsiveness after repeated exposure, so-called sensitization (Groves & Thompson, 1970). Responses that are subject to habituation or sensitization can include any output of the nervous system, thus affecting outputs ranging from physiology and hormonal release to subjective and behavioral responses. It is important to emphasize that these different levels of responses in turn can influence each other. For example, attenuated or increased hormone release can influence subjective and behavioral responses. Habituation and sensitization to approach–avoidance conflicts are well documented in several species (Bernstein & Nietzel, 1974; Erhard et al., 2006). Habituation and sensitization after one session of behavioral testing on the EPM have also been described in some rodent species (Treit et al., 1993), while they seem to be absent in others (Rico et al., 2016). For VR-based research in particular, little is known about potential habituation and sensitization effects for VR applications. Therefore, investigating the test–retest reliability of a VR application has great importance for VR research in general. However, research into approach–avoidance in VR differs significantly from clinical and therapeutic use of VR applications. The therapeutic effects of VR therapy are based on habituation effects. Therefore, therapeutic approaches rely on longer or more frequent exposures to allow habituation to occur (e.g., Gujjar et al., 2019). In contrast, in the present study, we used a long interval between exposures and exposed participants to the VR environment only for short periods. In an earlier study in rodents, an inter-trial interval of 28 days and change of the testing room was proposed to avoid habituation and sensitization on the EPM (Schneider et al., 2011). Here, we were interested in whether habituation and sensitization on the EPM could also be avoided in humans using a corresponding approach (28-day interval and change of the test surroundings). If test–retest profiles were stable, the EPM could be used repeatedly to measure approach and avoidance behavior. This would be a major methodological advantage, as repeated measures within subjects would improve statistical power in clinical and intervention studies. Moreover, it is of broad interest to better understand habituation and sensitization effects in VR research in general.

**Fig. 1:**
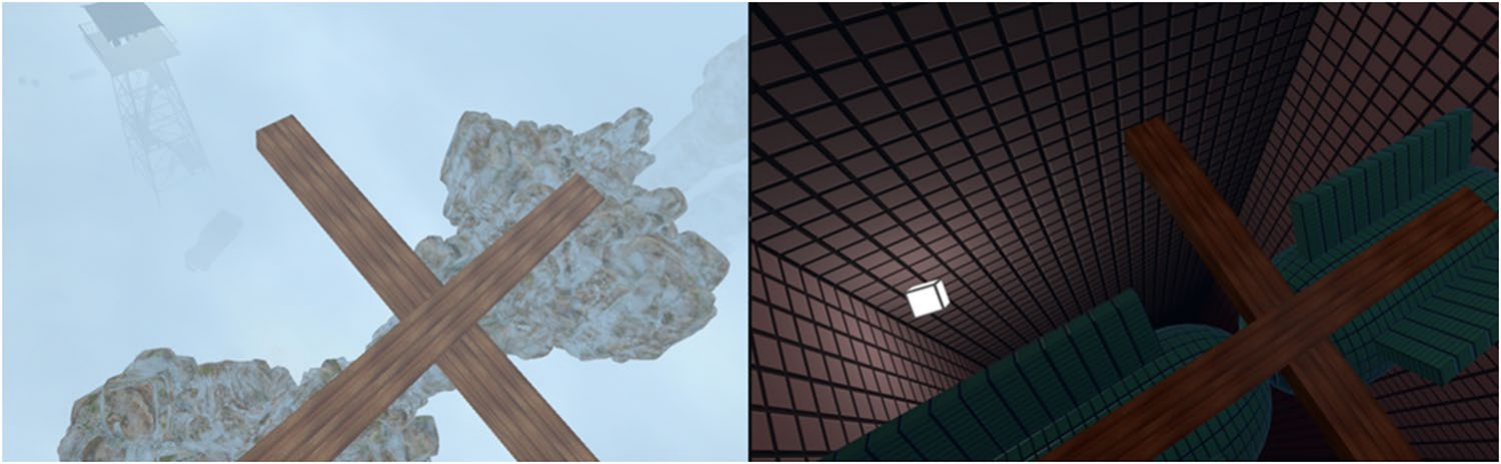
Two novel virtual reality environments for repeated exposure of the EPM: EPM_Desert_ (left) resembles a real-world situation, while EPM_VideoGame_ (right) shows abstract shapes reminiscent of a videogame. For videos please see https://osf.io/sbxpw/?view_only=870b2d3ca4da47ff803d64621e4b0f50; for pictures and a video of EPM_Sea_ please see Biedermann et al. (2017)

Earlier research suggested that VR can evoke lifelike responses at both the behavioral and psychophysiological levels and has the potential to increase the ecological validity of psychological experiments (Kisker, Gruber, & Schöne, 2021b). In our validated version of the human EPM, we tried to increase ecological validity with an augmented reality approach, e.g., by including a real wooden maze in a naturalistic scene above the sea (Biedermann et al., 2017). In the present study, we were also interested to explore whether a naturalistic setup is indeed needed to induce lifelike anxiety-related responses in humans. Thus, we designed two additional virtual environments for repeated exposure: one naturalistic desert scene (EPM_Desert;_ Fig. 1), and one video game-like scene including cubic patterns (EPM_VideoGame_; Fig. 1). These virtual environments were augmented using suitable sounds and wind from a ventilator (mixed-reality setup). All participants were first tested on the validated EPM (EPM_Sea_) and after 28 days randomly assigned to the other virtual environments to study the effect of repeated exposure and explore differences related to real-world resemblance.

## Methods

### Participants

Healthy individuals aged between 18 and 50 years were invited to participate through a variety of means, including a popular local website for biomedical research recruitment, word of mouth, and a notice at local universities. All participants were screened for somatic and psychiatric disorders as well as current drug use. Participants were informed about the procedure and gave written informed consent. All experiments were approved by the local ethics committee (Ärztekammer Hamburg, Germany), and the study was conducted according to good clinical practice guidelines as defined in the Declaration of Helsinki (2013).

Forty-four healthy participants (female = 22, male = 22) were invited into our laboratory twice. Five participants (11%) were lost at follow-up, resulting in a final sample of 39 participants (female = 19, male = 20). Mean age was 25.67 years ± 3.74 SD. The validated EPM_Sea_ was used for the first testing. Participants were then randomly assigned to the naturalistic EPM_Desert_ (*n* = 18) or to the video game-like EPM_VideoGame_ (*n* = 21) for the second testing 28 days after the first testing. We assessed anxious temperament with the Spielberger State-Trait Anxiety Inventory (STAI; Spielberger et al., 1970), and sensation seeking using the Sensation Seeking Scale (SSSV; Zuckerman et al., 1978). Gaming experience was assessed using a purpose-designed questionnaire (“ever played” and “how much regular gaming time” on computer games and virtual reality). After each exposure to the EPM, participants rated their anxiety level on the EPM on a scale from 0 (no anxiety) to 9 (very strong anxiety). The subjective experience of the virtual reality immersion was assessed using the iGroup presence questionnaire (Schubert et al., 1999), and side effects were measured using a simulator sickness questionnaire (Kennedy et al., 1993). All lab procedures were performed between 2 pm and 9 pm to avoid circadian influences on endocrinology.

### Human elevated plus-maze

The human EPM consists of a real-world wooden maze, which is simultaneously presented in virtual reality. The real-world maze consists of four wooden arms (width 30 cm, height 20 cm). Each arm has a length of 175 cm, covering in total 350 × 350 cm, within an experimental room (550 × 550 cm) with two virtual reality tracking systems (HTC Vive^®^ Base Station, Seattle, WA, USA) attached at 250-cm height at opposite walls. Participants entered the room with their eyes closed and were guided by one of the experimenters towards the maze. Participants received a head-mounted display (HTC Vive^®^, Seattle, WA, USA) and noise-canceling headphones (Bose QuietComfort^®^ 35, Framingham, MA, USA) and were instructed to open their eyes. After checking the vision of participants in a baseline graphical environment, the virtual reality software (EPM_Sea_, EPM_Desert_, or EPM_VideoGame_) was started. Participants found themselves in a 550 × 550 cm virtual room with a virtual wooden plus-maze in front of them. Importantly, the virtual reality plus-maze and the physical real-world plus-maze had the same shape, material, and size, as well as position in the virtual and real world. A recorded voice instructed participants to step on the maze and walk slowly towards the center of the maze, where participants had to wait for 60 s to allow for baseline measurements. Further, they were instructed that they would be allowed to explore the environment on the maze once the scene had changed. The behavioral experiment started after 90 s, and participants found themselves in a new environment (EPM_Sea_, EPM_Desert_, or EPM_VideoGame_). In the new scenario, only the virtual plus-maze remained unchanged. Instead of being in a virtual room, the maze was placed on a virtual rocky mountain surrounded by water (EPM_Sea_), or on a solid rock above a desert landscape (EPM_Desert_) or on futuristic bars in a video game-like vertical tunnel leading into a profound abyss (EPM_VideoGame_). Two opposite arms (closed arms) and the center of the maze were surrounded by rocks/tubes, while the other arms reached out over the water/desert/abyss (open arms). For further description and visualization, please see (Biedermann et al., 2017) and https://osf.io/sbxpw/?view_only=870b2d3ca4da47ff803d64621e4b0f50. Simultaneously with the change of the virtual environment, suitable sounds as well as two ventilators were started in the experimental room to increase presence in the virtual environment. The ventilators were placed at the end of the arm participants were initially facing to give the impression of a cool wind. Participants were invited to explore the EPM for 300 s. After the scenario ended, participants removed the head-mounted display and headphones and left the room with their eyes closed.

Data recording and synchronization of the EPM was performed as described previously (Biedermann et al., 2017): Before experimental testing, the virtual maze was synchronized with the real-world maze using a controller (HTC Vive^®^ controller, Seattle, USA), a purpose-built mounting device, and purpose-designed software (A+ cross^®^). During the alignment of the virtual environment and the physical maze, the calibration software (A+ cross^®^) aligned the axis of the 3D world space coordinate system with the arms of the physical maze. The world space coordinate system was created with its origin at the center of the maze, its *x*-axis aligned with the closed arms, and its *z*-axis aligned with the open arms of the plus-maze.

Headset position and orientation during a running experiment were sampled at 5 Hz for each sampling point i at time t to obtain a set of 3D positions ${\overrightarrow{p}}^i=\left({p}_x^i,{p}_y^i,{p}_z^i\right)$ and 3D orientations ${\overrightarrow{r}}^i=\left({r}_x^i,{r}_y^i,{r}_z^i\right)$.

These measurements were then analyzed by the software (A+ cross^®^) to evaluate participants’ movement patterns on the maze. A sample point pi counts towards time spent on one arm if the absolute position on one of the axes, and thus distance from center, is larger than a predefined threshold value. The total time spent on one of the open arms *t*_*o*_ and the closed arms *t*_*c*_ is calculated as ${t}_{o,c}=\sum {t}^i\left[|{p}_{x,z}^i|> threshold\right]$.

In addition to the time spent in the different areas of the maze, all movements of the headset were calculated as average velocities ${\overrightarrow{v}}_{o,c}$ whenever ${p}_{x,z}^i> thres{hold}_{\left[o,c\right]}$ and ${p}_{x,z}^{i-1}> thres{hold}_{\left[o,c\right]}$.

Sample points for which the inverse case, ${p}_{x,z}^i> threshold$ and ${p}_{x,z}^{i-1}< threshold$, was measured were counted towards the total number of entries *n*_[*o*, *c*]_ to one of the arms.

The following parameters were used for automated analyses: total time spent on open arms (time on open arms), number of entries of open arms, latency for the first entry of an open arm (latency first visit), and time until subjects reached the end of an open arm (latency endexploration). Moreover, time on and entries of closed arms were assessed as markers for locomotor activity.

### Heat maps

To visualize the length of stay on the EPM, heatmaps for the first and second exposure on the EPM were created. For this purpose, matrices *M*^*first*^ and *M*^*second*^ , each with a size of 35 × 35, represent 10 cm^2^ patches. Each entry *M*_*i*, *j*_ was incremented for each coordinate $$\left(\begin{array}{c}x\\ {}y\end{array}\right)$$ with $i<\left\lfloor \frac{x}{10}\right\rfloor <i+10$ and $j<\left\lfloor \frac{y}{10}\right\rfloor <j+10$. Finally, the values were normalized by dividing the values by the number of participants. For the difference matrix *M*^*difference*^ , *M*^*first*^ was subtracted from *M*^*second*^.

### Psychophysiology

Skin conductance levels (SCL), respiratory rate, and heart rate (electrocardiogram, ECG) were recorded using BioNomadix wireless physiology devices and a BIOPAC MP150 data acquisition system and were analyzed using AcqKnowledge 4.4.1 software (Biopac Systems, Goleta, CA, USA). Skin conductance Ag/AgCl electrodes were attached to the palm of the nondominant hand 10 min before behavioral testing. Baseline levels were recorded 30 s before behavioral testing, and average levels were compared to average levels of 30 s intervals during behavioral testing, leading to 11 intervals (baseline, 0–30 s, 30–60 s, etc.). At first testing, four SCL datasets (10%) had to be excluded, and at second testing, nine SCL (23%), two respiration (5%), and two ECG (5%) datasets had to be excluded due to poor data quality, for example, due to movement artifacts and electrode disconnection.

### Salivary alpha-amylase

Saliva sample collection was performed before (T0), directly after (T1), and 15 min (T2) after behavioral testing. Participants received oral instructions on the correct use of the Salivette salivary collection device (Sarstedt AG Nümbrecht, Germany). Samples were centrifuged and saliva stored at −80° C until further analysis. α-Amylase was determined using a commercial liquid-phase enzymatic assay (RE80111, IBL International, Hamburg, Germany). Intra- and inter-assay coefficients of variance were below 7%, and the detection limit was 25 U/mL.

### Statistical analyses

Statistical analyses were carried out using R, version 4.0.2 (http://www.r-project.org/) and IBM SPSS Statistics 22.0 (IBM Corp., Armonk, NY, USA). An a priori power analysis was conducted using G*Power 3 (Faul et al., 2007) to detect a significant difference for the factor time on open arms in a paired *t*-test with a medium effect size (*d* = .50) and an alpha of .05. Results showed that a total sample size of 38 participants was required to achieve a power of .85.

Pearson correlations and intra-class correlations (ICC) (model: two-way mixed, type: consistency) were computed to assess test–retest reliability.

Psychophysiological data were analyzed using repeated-measures analysis of variance (ANOVA) with the factors *time*, e.g., the 11 intervals (baseline, 0–30 s, 30–60 s, etc.), and *repeated exposure* (first and second testing) as within-subject factors and *EPM version* as between-subject factor. For post hoc testing, we compared baseline and the first interval (0–30 s) with paired *t*-tests. SCL data were transformed to logarithmic values (logarithmus naturalis [ln]). Alpha-amylase levels were analyzed using repeated-measures ANOVA with the factors *time* (T0–T2) and *repeated exposure* (first and second testing) as within-subject factors and *EPM version* as between-subject factor. Subjective ratings and measures of anxiety-related behavior were analyzed using repeated-measures ANOVA with the *repeated exposure* (first and second testing) as within-subject factors and *EPM version* as between-subject factor. Statistical significance was set at *p* < 0.05. Effect sizes are given as η2 partial for ANOVAs. We deliberately omitted corrections for multiple testing because this would have decreased the chance of finding statistically significant differences between the different EPM versions.

## Results

Due to the randomization procedure, we assumed that sociodemographic parameters including video game and virtual reality experience, as well as trait anxiety and sensation seeking, were evenly distributed. Therefore, these potential confounders were not considered in the following analyses.

### Anxiety-related behaviors are stable at repeated exposure

We found no significant effect of *repeated exposure* on the core behavioral outcome variables of the EPM, i.e., time on open arms (*F*_1,37_ = 1.80, *p* = 0.188, η2_partial_ = 0.046), number of entries of open arms (*F*_1,37_ = 1.21, *p* = 0.278, η2_partial_ = 0.032), latency open arm exploration (*F*_1,37_ = 0.66, *p* = 0.422, η2_partial_ = 0.018), and latency open arm endexploration (*F*_1,37_ = 3.21, *p* = 0.081, η2_partial_ = 0.080; Table 1).

**Table 1:** Mean and standard deviation (SD) of the core behavioral markers on the EPM

Behavioral measures	First exposure Mean (SD)	Second exposure Mean (SD)	EPM_VideoGame_ Mean (SD)	EPM_Desert_ Mean (SD)
Total entries open arms [n]	4.1 (2.6)	3.6 (2.3)	3.5 (2.3)	3.8 (2.2)
Latency open arms [s]	73.5 (98.6)	85.8 (88.1)	87.0 (96.9)	84.5 (79.5)
Latency endexploration [s]	180.6 (119.5)	211.6 (110.8)	191.9 (124.2)	234.6 (91.0)
Time on open arms [s]	91.0 (56.1)	81.2 (50.2)	79.6 (58.5)	83.2 (40.1)
Total distance covered [m]	13.4 (5.9)	12.6 (4.8)	13.0 (4.8)	12.2 (5.0)

Other behavioral measures that reflect locomotor activity such as the total distance covered on the EPM (*F*_1,37_ = 1.75, *p* = 0.194, η2_partial_ = 0.045) and the average velocity on closed arms (*F*_1,37_ = 0.31, *p* = 0.581, η2_partial_ = 0.008) were also not significantly different between baseline and second testing. Moreover, *EPM version* (EPM_VideoGame_ vs. EPM_Desert_) did not significantly affect behavior (all *p* > 0.3).

**Fig. 2:**
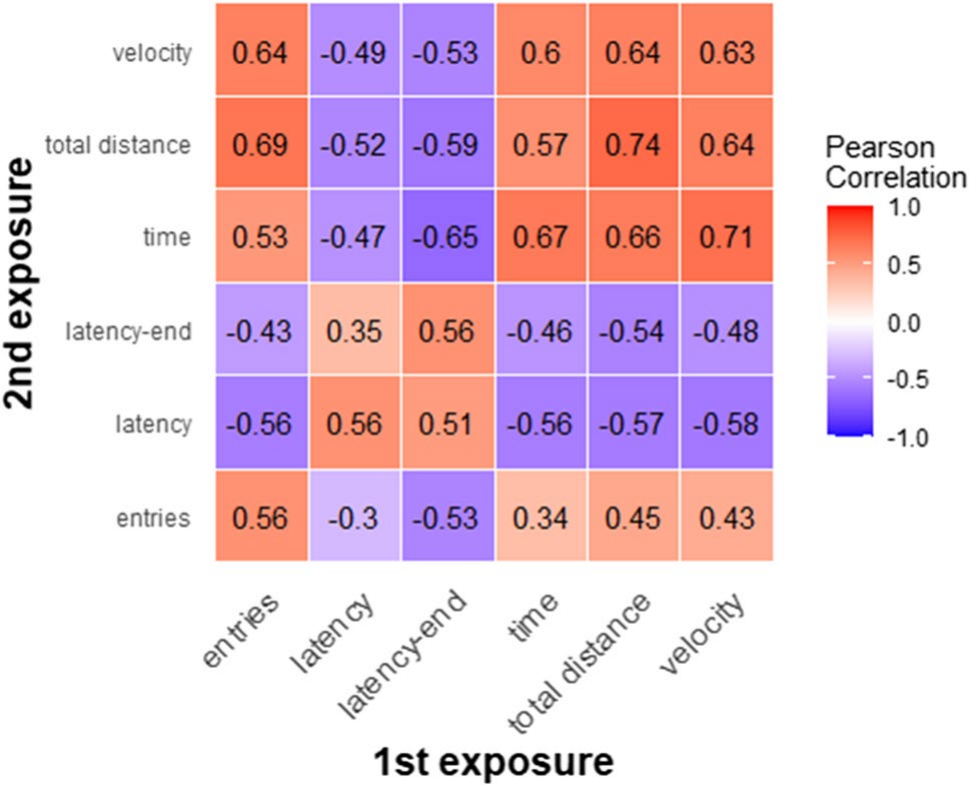
A correlation matrix heatmap of behavioral measures of the EPM at first and second exposure. Numbers represent correlation coefficients. Four measures of anxiety-related behavior: entries = total entries open arms, latency = latency open arms, latency-end = latency endexploration, time = time on open arms; as well as two measures of locomotion: total distance = total distance covered, velocity = average velocity on closed arms

Anxiety-related behavior showed a high correlation between baseline and repeated exposure to the EPM (Fig. 2; all *p* < 0.001). To assess test–retest reliability for the behavioral measures, we calculated ICC for all behavioral measures. In reliability studies, an ICC > 0.7 is commonly used as a threshold of “sufficient reliability” (Hripcsak & Heitjan, 2002), and all ICC for behavioral measures in the present study were above this threshold (entries = 0.72 [95% CI = 0.46–0.85], latency = 0.71 [95% CI = 0.45–0.85], latency-end = 0.72 [95% CI = 0.46–0.85], time = 0.80 [95% CI = 0.62–0.89], total distance = 0.84 [95% CI = 0.69–0.92], velocity = 0.79 [95% CI = 0.60–0.89]).

**Fig. 3:**
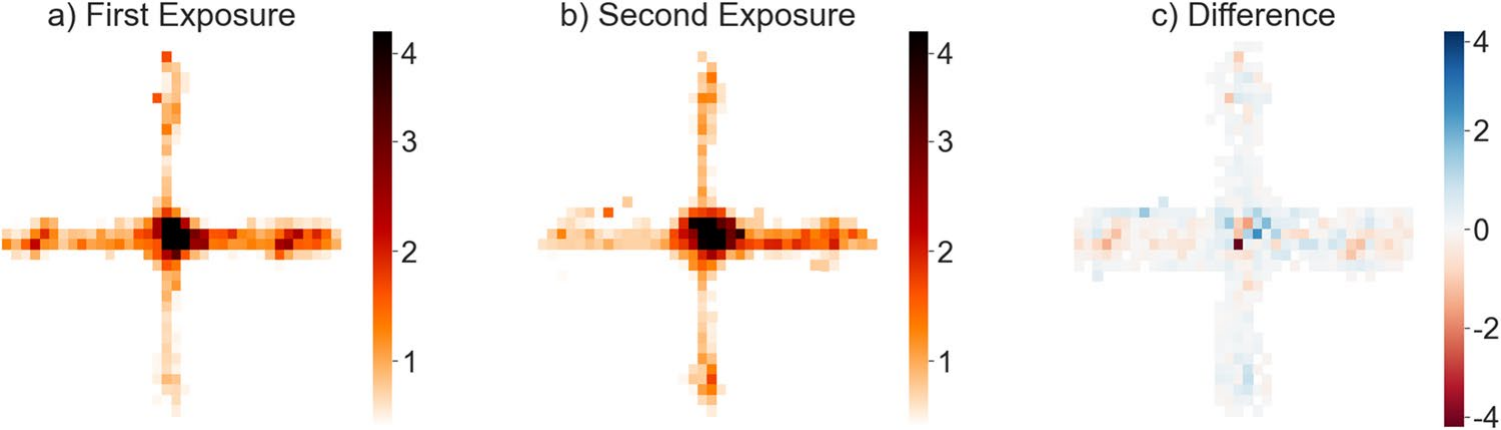
Heatmaps indicate length of stay of at least 0.5 s for the first (**a**) and second (**b**) exposure on 10 cm^2^ areas. Darker values indicate a longer average length of stay in that area. **c** Differences in mean length of stay between the first and the second measurement. Red hues indicate that participants spent less time in an area, and blue hues indicate that participants spent more time in an area on the second exposure

Visual inspection of the heatmaps showed no obvious differences between the two time points (Fig. 3).

### The influence of repeated exposure to the EPM on autonomic reactions

Exposure to the EPM led to an activation of the autonomous nervous system. *Repeated exposure* to the EPM, however, changed the pattern of autonomic response for some of the markers. As expected, salivary alpha-amylase levels increased directly after exposure to the EPM, which is reflected in a significant effect of *time* (*F*_2, 74_ = 5.278, *p* = 0.007, η2_partial_ = 0.125). R*epeated exposure* and *EPM version* had no effect on the salivary alpha-amylase response (all *p* > 0.3). Post hoc comparison showed a significant increase in salivary alpha-amylase levels between T0 and T1 at first (*t* =−3.62, *df* = 38, *p* = 0.001) and repeated exposure (*t* = −2.25, *df* = 38, *p* = 0.03).

**Fig. 4:**
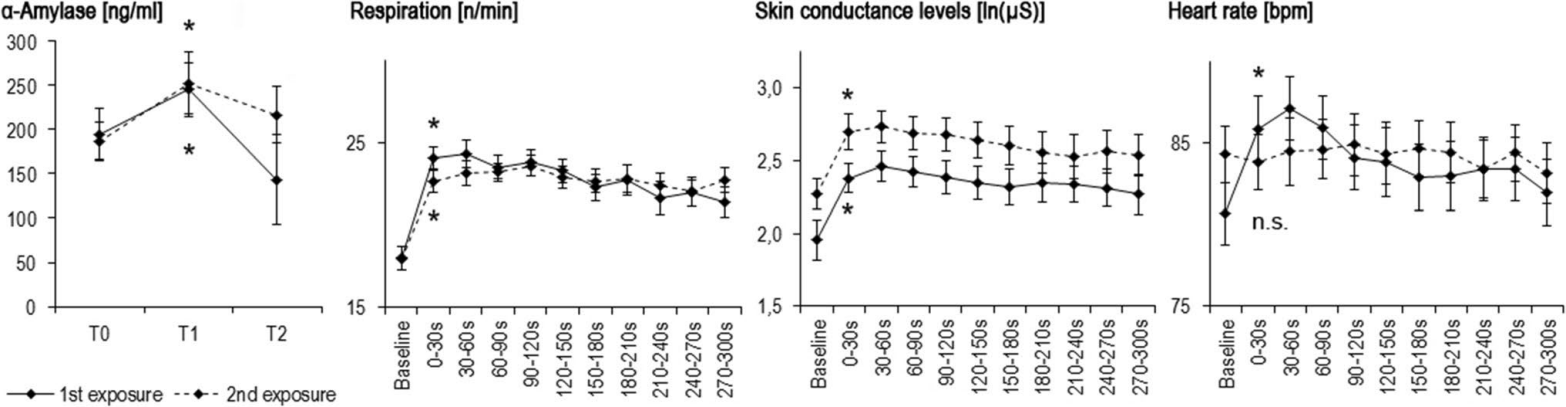
Repeated exposure to the EPM and autonomic responses. T0 = before, T1 = directly after and T2 = 15 min after EPM. Data points represent means ± SEM. * represents a significant difference between T0 and T1 or baseline and 0–30 s in post hoc tests. n.s. is not significant for the post hoc comparison between baseline and 0–30 s at second exposure

On a psychophysiological level, respiratory frequency (main effect of time: *F*_6.53, 216.64_ = 16.88, *p* < 0.001, η2_partial_ = 0.345) and skin conductance levels (main effect of time: *F*_2.52, 83.28_ = 17.41, *p* < 0.001, η2_partial_ = 0.431) increased with *time*. *Repeated exposure* had no effect on respiratory rate (*p* > 0.3), but increased skin conductance levels (*F*_1, 83.28_ = 4.98, *p* = 0.036, η2_partial_ = 0.178). While heart rate also showed a significant effect of *time* (*F*_5.26, 202.39_ = 3.68, *p* < 0.001, η2_partial_ = 0.103) in the repeated-measures ANOVA, the pattern was different at repeated exposure, which is reflected in a significant *repeated exposure* * *time* interaction (*F*_6.33, 202.39_ = 3.52, *p* = 0.002, η2_partial_ = 0.099). Post hoc testing revealed a significant increase in psychophysiological levels between baseline and onset of the experiment (0–30s) for heart rate (*t* = −4.52, *df* = 38, *p* < 0.001), respiratory rate (*t* = −8.27, *df* = 38, *p* > 0.001), and skin conductance levels (*t* = −6.61, *df* = 36, *p* < 0.001) at first exposure. At repeated exposure an increase was found only for respiratory rate (*t* = −7.29, *df* = 36, *p* > 0.001) and skin conductance levels (*t* = −8.51, *df* = 29, *p* < 0.001). Heart rate (*t* = 0.36, *df* = 36, *p* = 0.72) showed no change between baseline and onset of behavioral testing at repeated exposure. Interestingly, at repeated exposure, baseline heart rate levels were descriptively higher than at first exposure and showed no response after onset of EPM testing (Fig. 4). *EPM version* had no significant effect on psychophysiological measures (all *p* > 0.3).

### The subjective response to repeated EPM exposure

**Fig. 5:**
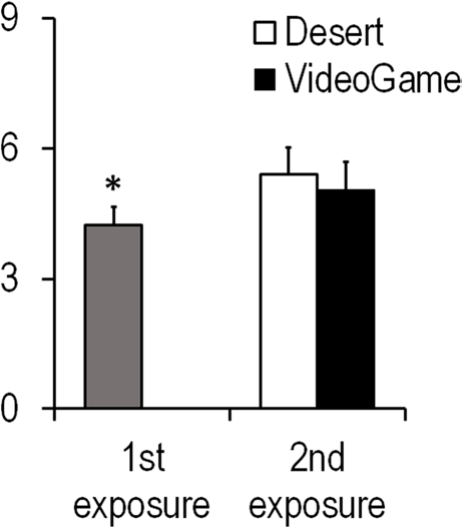
Subjective anxiety levels at first and second exposure to the EPM. Bars represent means + SEM, * *p* < 0.05

Spatial presence (F_1,37_ = 0.26, *p* = 0.67, η2_partial_ = 0.007), emotional involvement (*F*_1,37_ = 2.25, *p* = 0.14, η2_partial_ = 0.057), and perceived realism (*F*_1,37_ = 0.005, *p* = 0.94, η2_partial_ < 0.001) (iGroup presence questionnaire) as well as total side effects (*F*_1,37_ = 2.65, *p* = 0.11, η2_partial_ = 0.067) (simulator sickness questionnaire) were comparable at first and second exposure to the EPM. They were also comparable between EPM_VideoGame_ and EPM_Desert_ (spatial presence *t* = 0.13, *df* = 37, *p* = 0.90; emotional involvement *t* = −0.49, *df* = 37, *p* = 0.63, perceived realism *t* = −0.74, *df* = 37, *p* = 0.94, total side effects *t* = 1.26, *df* = 37, *p* = 0.22). However, participants reported higher levels of anxiety at repeated exposure to the EPM (*F*_1,37_ = 6.19, *p* = 0.018, η2_partial_ = 0.143; Fig. 5). No significant difference in subjective anxiety between EPM_VideoGame_ and EPM_Desert_ was found (*p* = 0.71). Self-reported anxiety levels at baseline and second testing correlated strongly with each other (*r* = 0.61, *p* < 0.001).

## Discussion

The main finding of this study is that repeated testing of anxiety-related behavior on the human EPM can be reliably carried out using the current protocol. We found high correlations between the same behavioral measures and across different measures comparing first and second testing, indicating that approach–avoidance behavior on the EPM is a stable measure. Moreover, certain physiological markers also seem to be stable over time, as we found that the characteristic increases in respiratory frequency and α-amylase levels - both indicators of autonomic activation - were comparable at repeated exposure. However, this was not the case for heart rate, skin conductance levels, or subjective anxiety ratings. Interestingly, heart rate no longer showed the characteristic increase between baseline and the beginning of testing at repeated exposure and instead was elevated before the second testing began, whereas subjective anxiety levels and skin conductance levels increased significantly at repeated exposure. Both effects may be explained by anticipatory anxiety before the second testing as well as sensitization. The anticipation of aversive situations involving novelty and unpredictability has been shown to be associated with the release of catecholamines (Mason, 1968) and an increase in SCL (Epstein & Roupenian, 1970). Markers that are sensitive to catecholamine release and have good temporal resolution (as opposed to alpha-amylase levels collected at only three time points) are particularly sensitive to anticipatory anxiety directly before onset of the task. It has been shown that this is the case for heart rate and skin conductance levels (Armario et al., 2020; Boesch et al., 2014). During the baseline condition at repeated exposure, participants knew that they would again be standing on a wooden cross that had been followed by an anxiety-related environment during the first test. Thus, it is most likely that the elevated heart rate in the 30 s before the onset of the second EPM testing reflects anticipatory anxiety (Butler & Mathews, 1987). The elevation in skin conductance levels during the second EPM testing could also result from anticipatory anxiety. However, from our understanding, anticipatory anxiety relates rather to outcomes that can be measured before a certain event, whereas sensitization effects reflect a general sensitization of psychophysiological or subjective markers of anxiety during an event. The observed higher subjective anxiety levels and higher skin conductance levels throughout the experiment at repeated exposure might thus also represent a form of sensitization. As hypothesized, our analyses showed no indication of habituation to the EPM at repeated exposure. This is in line with the hypothesis that very strong stimuli do not show a significant response decrement, particularly after only one exposure repetition and a certain interval between exposures (Rankin et al., 2009). The EPM thus differs from other behavioral tasks that elicit a stress response, such as the Trier Social Stress Test, which shows habituation of subjective ratings at repeated exposure (Boesch et al., 2014; Cohen et al., 2000; Jönsson et al., 2010) but no habituation of other markers such as the autonomic stress response (Gerra et al., 2001; Jönsson et al., 2010). However, we found indications of a possible sensitization effect at repeated exposure. Therefore, we suggest using a crossover design (see e.g., Siebers et al., 2021) for repeated testing with the EPM (e.g., to test a certain intervention or compound compared to a placebo treatment). Repeated testing, such as for a placebo-controlled treatment trial, should be reliable as long as all respective parameters are evaluated in one statistical model (e.g., treatment as interindividual and first and second testing as intraindividual effects within analysis of covariance [ANCOVA]). For the time being, we do not recommend repeated testing without a placebo group.

To our knowledge, this study represents the first investigation of test–retest reliability of a VR-based setting. By using a between-testing interval of 28 days and novel virtual environments at repeated exposure, we were able to avoid habituation effects, which otherwise could have influenced the affective effects in VR. Other VR research could therefore benefit from taking this into account when designing studies to minimize possible habituation effects. However, other factors that could lead to habituation, such as participants' VR or gaming experience, should be further investigated to draw more general conclusions for habituation effects in VR research. Possible sensitization and anticipatory anxiety effects might have been at play in our study, altering the pattern of autonomic response for heart rate and skin conductance levels. Sensitization and anticipation could therefore also affect other VR-based research. At least in the case of a repeated-measures study design, this should be considered. However, it is also possible that these observed effects occur mainly in the context of aversive (frightening) VR applications, such as the one we used. Further studies with repeated measures examining sensitization and anticipation in other VR contexts (e.g., using relaxing or calming VR environments) are therefore much needed.

We found that the EPM environments were able to reliably test anxiety-related behaviors as well as their underlying physiological and subjective correlates. Therefore, our results are in line with other studies which showed that virtual heights consistently evoke subjective, behavioral, and psychological fear responses across different setups (Asjad et al., 2018; Biedermann et al., 2017; Gromer et al., 2018, 2019; Kisker et al., 2019a).

The feeling of presence together with immersivity is considered to be crucial for inducing affect and thus for the real-life experience in VR (Felnhofer et al., 2015; Kisker, Gruber, & Schöne, 2021b; Kisker, Lange, et al., 2021a; Slater & Wilbur, 1997). It has been proposed that presence levels differ under different emotional states (Baños et al., 2004, 2008; Riva et al., 2007). However, a VE inducing boredom was found to elicit as much of a feeling of presence as other emotional states induced by a VE (e.g., joy or anxiety; Felnhofer et al., 2015). Thus, it does not seem to be crucial how interesting and captivating a VR is for it to evoke the feeling of presence. Instead, the feeling of presence might be a prerequisite for any emotional affect to occur in VR (Felnhofer et al., 2014), although experimental evidence is inconclusive (Gromer et al., 2018, 2019). Similarly, it has been suggested that a VE should be as realistic as possible to create an advanced simulation of reality, e.g., by using 3D photos and videos instead of computer-generated VEs, which are easily recognized as unreal (Schöne et al., 2021). Yet, our results show that the two new EPM versions (EPM_Desert_, EPM_VideoGame_) did not differ significantly in their reliability for testing anxiety-related behaviors and their physiological and subjective correlates. This indicates that resemblance to real life is not that critical for ecological validity of the human EPM given that presence and immersion in the virtual environments are comparable. This may also be facilitated by the mixed-reality design of both EPM versions. An increase in immersion achieved by adding tactile cues (i.e., wind simulation) was found to enhance VR-triggered fear responses in a virtual height scene (Gromer et al., 2018). Increasing immersion by using haptic cues in a virtual spider simulation (Hoffman et al., 2003; Peperkorn & Mühlberger, 2013) or stereoscopy in a virtual height simulation (Mühlberger et al., 2012) resulted in an increased feeling of presence and fear. Thus, the sensorimotor interactions in a mixed-reality setup may contribute sufficiently to the VR experience in a computer-generated VE to give participants the impression of being directly affected by the VE (Gromer et al., 2018; Kisker, Gruber, & Schöne, 2021b; Kisker, Lange, et al., 2021a; Slater & Wilbur, 1997). We consider the result of our study showing no significant difference in testing of both EPM versions to be an important finding. It is technically much easier to develop video game-like scenarios, given that less effort is needed to design them as close to reality as possible. However, it seems to be important to keep the core characteristics of the virtual EPM (e.g., open arms above life-threatening height) constant.

### Limitations

It would have been helpful to assess anticipatory anxiety before repeated exposure to the EPM to confirm our hypothesis that the characteristic autonomic activation during repeated exposure was absent in some parameters due to anticipatory anxiety. Moreover, it is important to stress that we used only two time points for repeated testing, with a 4-week interval; the likelihood for habituation effects on the core behavioral measures reflecting anxiety-related behaviors increases with more repetitions and comparable environments (Schrader et al., 2018). We did not compare different time intervals between the two measurements. It is possible that shorter or longer intervals lead to similar results and are comparably valid. Also, we did not compare our new VR environments to the reuse of the EPM_Sea_; therefore, we cannot conclude that the use of a novel VR environment at second exposure is necessary.

In the future, it would be interesting to study the hypothalamus-pituitary-adrenal (HPA) axis response to repeated exposure to the EPM. Earlier research indicates that the HPA axis habituates to repeated exposure, while the autonomic response does not (Gerra et al., 2001; Schommer et al., 2003; Wüst et al., 2005), and thus salivary cortisol is more sensitive to endocrine habituation than salivary α-amylase levels. Although we assessed gaming experience among the participants, the lack of difference within our sample did not allow for any conclusions about possible habituation effects related to gaming experience. The impact of gaming experience on affective effects of VR is one of the most important issues that VR research has yet to deal with. On that account, future studies should focus on including participants with a wide range of gaming experiences to investigate possible habituation effects in VR.

## Conclusion

To maintain the efficacy of the EPM in provoking anxiety and eliciting anxiety-related behavior at repeated exposure, we advise the use of the protocol presented in this paper (i.e., a between-testing interval of 28 days and the use of novel virtual environments at repeated exposure). Our data suggest that one repetition of behavioral testing on the human EPM is reliably possible under such circumstances, while real-world resemblance seems to play a minor role for this test.

## Data Availability

The data and materials for all experiments are available at https://osf.io/sbxpw/?view_only=870b2d3ca4da47ff803d64621e4b0f50 and at https://bmcbiol.biomedcentral.com/articles/10.1186/s12915-017-0463-6#Sec18 (Biedermann et al., 2017; Video of EPM_Sea_). None of the experiments was preregistered.
